# Automated multi-model framework for malaria detection using deep learning and feature fusion

**DOI:** 10.1038/s41598-025-04784-w

**Published:** 2025-07-16

**Authors:** Osama R. Shahin, Hamoud H. Alshammari, Raed N. Alabdali, Ahmed M. Salaheldin, Neven Saleh

**Affiliations:** 1 Department of Computer Science, College of Computer and Information Sciences, Jouf University, Sakaka, Saudi Arabia; 2 Department of Information Systems, College of Computer and Information Sciences, Jouf University, Sakaka, Saudi Arabia; 3 Biomedical Engineering and Systems Department, Faculty of Engineering, Cairo University, Giza, Egypt; 4 Biomedical Engineering Department, Faculty of Engineering and Technology, Future University in Egypt, New Cairo, Egypt

**Keywords:** Malaria detection, AI solutions, Feature fusion, CNN, Majority voting, Infectious diseases, Disease prevention, Health services

## Abstract

Malaria remains a critical global health challenge, particularly in tropical and subtropical regions. While traditional methods for diagnosis are effective, they face some limitations related to accuracy, time consumption, and manual effort. This study proposes an advanced, automated diagnostic framework for malaria detection using a multi-model architecture integrating deep learning and machine learning techniques. The framework employs a transfer learning approach that incorporates ResNet 50, VGG16, and DenseNet-201 for feature extraction. This is followed by feature fusion and dimensionality reduction via principal component analysis. A hybrid scheme that combines support vector machine and long short-term memory networks is used for classification. A majority voting mechanism aggregates outputs from all models to enhance prediction robustness. The approach was validated on a publicly available dataset comprising 27,558 microscopic thin blood smear images. The results demonstrated superior performance, achieving an accuracy of 96.47%, sensitivity of 96.03%, specificity of 96.90%, precision of 96.88%, and F1-score of 96.45% using the majority voting ensemble. Comparative analysis highlights the framework’s advancements over existing methods in diagnostic reliability and computational efficiency. This work underscores the potential of AI-driven solutions in advancing malaria diagnostics and lays the foundation for applications in other blood-borne diseases.

## Introduction

Red blood cell (RBC) assessment is a significant diagnostic tool for a wide range of diseases, including anaemia, thalassemia, and malaria^1–3^. When morphological characteristics of the RBC are altered, this change may indicate an infection with a parasite, such as malaria. Usually, blood diseases are diagnosed based on physical examinations of the blood. However, microscopic images play a pivotal role in diagnosing such illnesses. Incorporating the counting of RBCs and examining the morphological change of the cell shape may result in a more comprehensive diagnosis.

Malaria is a global health challenge that leads to deaths, particularly among young children and pregnant women. It is a mosquito-borne disease that is widely spread in tropical and subtropical regions. The common cause of the disease is a Plasmodium parasite that infects humans through female Anopheles mosquitoes^4–6^. In 2022, the World Health Organization (WHO) reported that 96% of malaria cases were recorded in twenty-nine countries globally. It was estimated that there were 249 million malaria cases, with a frequency of cases that was 58 out of 1000 individuals at high risk^7^. In Africa, malaria cases contributed to 90% of global cases and 92% of malaria-related deaths^8^.

Typically, malaria symptoms are similar to many diseases, like the flu, causing a strong fever, sweating, and headache. Additionally, it manifests with nausea, vomiting, and muscular consequences^9^. If the disease is left without appropriate therapy or ineffective drugs, it leads to grave consequences, specifically for children and pregnant women. The severity includes damage to the RBCs, which leads to severe anaemia and dysfunction of the respiratory system^1^. Besides, it impacts the musculoskeletal functions and cardiac muscles, which might result in serious complications^9^.

Microscopic medical image analysis has significantly contributed to the identification and classification of numerous blood-type illnesses. Examining these images manually can lead to an inaccurate diagnosis. Globally, almost all microscopic images are prepared as thin blood smears and digitally as thick films^1,10,11^. Typically, malaria is one of the blood-borne illnesses that is commonly diagnosed via microscopic images. Generally, the peripheral blood smears, known as the Giemsa-stained blood films, are the gold standard for diagnosing malaria. This technique involves the visual examination of thick and thin blood films. The aim of inspecting stained, thick blood smear slides under a microscope light is to detect and quantify parasites. Thin blood smear slides are used for identifying species of parasites^1,11^. In diagnosis, the degree of morphological changes of the RBC indicates the severity of malaria. The changes entail size variation, distribution variation, and shape variation^4,11^. Infected RBCs appear in light red, while parasites appear in blue and dark red colors^1^.

However, this manual technique presents several significant challenges that motivate the exploration of automated Artificial Intelligence (AI)-driven solutions. Firstly, accurate diagnosis demands a high level of expertise and extensive training, as the morphological changes in RBCs must be carefully assessed to determine the severity of infection. Furthermore, the visual distinction between malaria parasites and other cellular artifacts or parasitaemia can be highly ambiguous, increasing the risk of misdiagnosis. Another major limitation lies in the labor-intensive nature of manual slide examination^1,2^. To confidently declare a negative result, a specialist must thoroughly inspect at least 200 high-powered fields without detecting any parasites^6^. This process is time-consuming and susceptible to human error, particularly in high-volume or resource-limited clinical settings. Additionally, the consistency and reproducibility of manual diagnosis can vary significantly among different operators and laboratories. Such variability can impact the timeliness and effectiveness of treatment, especially in endemic regions where rapid decision-making is crucial.

Given these challenges, there is a clear need for more efficient, accurate, and scalable diagnostic solutions. In recent years, AI techniques – particularly machine learning (ML) and deep learning (DL) – have had a significant impact in this domain^12^. These methods address several limitations, such as inaccuracy, time consumption, and the extensive manual effort required^2^. Moreover, AI offers the capability to analyze a larger number of images in a much shorter time.

One major advantage of AI is its ability to automate the detection of malaria, including identifying the presence, type, and severity of parasites^4^. Since AI-based detection and classification are generally faster and more consistent than manual methods, they contribute to more reliable diagnosis and standardized treatment protocols. As a result, the application of various AI techniques in malaria diagnosis has seen considerable growth. In this context, both ML and DL algorithms are being widely applied to support a range of tasks related to malaria detection and analysis^3^. The key contributions of this study are as follows:

- **Development of a hybrid classification framework**: A novel diagnostic framework is proposed by integrating multiple DL architectures – ResNet-50, VGG-16, and DenseNet-201 – with traditional classifiers, such as Support Vector Machine (SVM) and Long Short-Term Memory (LSTM) networks. This approach leads to improved diagnostic accuracy of malaria detection.
- **Implementation of feature fusion for optimal representation**: The study introduces a feature fusion strategy that effectively combines and reduces high-dimensional features extracted from different DL models. This process eliminates redundant or irrelevant features while preserving the most informative ones, resulting in a discriminative feature vector.
- **Application of majority voting mechanism for robust decision-making**: A majority voting ensemble method is employed to integrate predictions from the various classifiers. This strategy ensures a more stable and reliable final decision by reducing the likelihood of misclassification due to individual model bias or overfitting.
- **Comprehensive performance evaluation using multiple metrics**: The proposed approach is rigorously evaluated using a variety of performance metrics, including accuracy, precision, recall, F1-score, and AUC. This ensures the generalization of performance across different scenarios.
- **Comparative analysis with existing methods**: The results obtained from the proposed system are compared with those of other state-of-the-art methods in the literature. The hybrid framework demonstrates superior performance, achieving higher classification accuracy and greater diagnostic reliability, proving the advantage of integrating DL with ML classifiers.
- **Contribution to AI-driven healthcare diagnostics**: This study not only advances the field of malaria detection but also contributes to the broader domain of AI applications in healthcare. By benchmarking and validating a robust, automated diagnostic pipeline, the research lays the groundwork for future developments in intelligent disease diagnosis systems.

The rest of this article is organized as follows. Related studies on malaria detection using AI methods are covered in the Related Works section. Before presenting the adopted methodology, we provide a Background section to describe the techniques utilized. The Materials and Methods section introduces the proposed pipelines using machine learning and deep learning algorithms. The results of the adopted methods are addressed in the Results section. The Discussion section explains the significance of the results and compares them with those of other related works. Finally, the Conclusion section summarizes the study and outlines expectations for future work.

## Related works

Numerous studies have been conducted for malaria diagnosis using a range of AI algorithms. Despite malaria still being diagnosed with traditional techniques, including rapid diagnostic (RD) and the polymerase chain reaction (PCR) tests^3,13^, AI algorithms remain promising. Convolutional neural networks (CNNs), particularly with diverse architectures and integration with various techniques, demonstrate meticulous detection of malaria. Moreover, microscopic images are widely used for investigation, either in thin or thick smear film forms.

A series of ML models were employed to forecast malaria, including random forest (RF), artificial neural network (ANN), multiple linear regression, and adaptive neuro-fuzzy inference system^3^. This study used haematological dataset belonging to 2207 Ghanaian patients with only twenty features. The results were assessed by statistical metrics, named R, R^2^, MSE, and RMSE. The ANN model demonstrated the best performance among the other models, while the RF was the worst model. A set of RBCs was used by Sunarko et al.^1^ to detect malaria. The work proposed Otsu’s method to segment the cells and distinguish the background and cell boundary from malaria parasites. Statistical features and skewness were used to detect the healthy cells from those infected. A total of 2000 thin blood smear images were used for training, and another 2000 microscopic images were used for testing. Both the grayscale images and RGB images were analyzed to extract the intensity distributions. Additionally, a K-means clustering method was employed to categorize the different colors within the cell, including identifying the parasites. The results revealed that there was 94.60% accuracy in detecting the infected cells.

Muhammed et al.^10^ study focused on identifying the rouleaux formation morphology of the RBCs caused by malaria parasites. Two CNN models were used with different sizes of input images. The study revealed the optimal image size was 300 × 300, which yielded an accuracy of 90.95% for detecting abnormal rouleaux formation. An extension of that work was presented in^11^, where five CNN models were employed for a dataset obtained from 100 infected patients. This includes XceptionNet, ResNet 50, DenseNet, EfficientNetB4, customized CNN, achieving accuracies of 96.40%, 98.50%, 99.00%, 96.20%, and 98.40%, respectively. Kassim et al.^14^ have conducted a study based on real curated data in Bangladesh. They used a total of 965 microscopic thin images belonging to 193 patients. Additionally, the dataset was divided into a polygon set and a point set to discriminate training and testing images, respectively. The authors presented RBCNet by using U-Net to segment the RBCs and then applying faster R-CNN to distinguish infected and non-infected RBCs. The precision for the tested images (point set of data) was 97.54 ± 1.44.

A comprehensive framework was developed in^2^ to identify the RBCs in microscopic images. To accurately identify areas of interest, RBCs were efficiently characterized using a region-based CNN model. Hoyos and Hoyos developed a deep learning-based model using YOLOv8 to detect malaria in addition to leukocytes, based on thick smear microscopic images. The study concluded that the developed model presented promising results compared to other related work, achieving an accuracy of 98.00% and 95.00% in detecting leukocytes and malaria parasites, respectively^6^. Another relevant study focused on using various machine learning models to diagnose malaria^3^. The applied algorithms were ANN, multi-linear regression, adaptive neuro-fuzzy inference system, and random forest. All models were trained based on fifteen criteria related to hematological parameters of blood tests, such as RBC count, hemoglobin level, etc. The mean square error, root mean square error, R^2^ coefficient, and R coefficient were used to characterize the malaria predicting results.

In 2023, automated CNN models were developed to diagnose malaria^13^. The authors employed a customized CNN, ResNet50, and MobileNetV2. A dataset consisting of 27,558 smear images with 50% infected images and 50% free parasitized images was investigated. The CNN model detected malaria with an accuracy of 95.50%, the MobileNetV2 has achieved an accuracy of 97.06%, and the ResNet50 yielded an accuracy of 96.70%. In this way, the MobileNetV2 consistently outperformed in diagnosing malaria. The question of whether thin smear images or thick smear images are most appropriate for malaria detection was answered by Ozsahin et al.^15^. In this context, a customized CNN model was applied to a dataset containing infected images with *P. falciparum* and *P. vivax* in addition to uninfected images. Moreover, a transfer learning approach has been adopted to validate the model by using the VGG16, ResNet50, and Inception V3. The evaluation metrics, including accuracy, precision, sensitivity, and F1-score, demonstrated strong performance for thick smear images. Therefore, the study revealed that the thick smear images outperformed the thin smear images in malaria diagnosis.

Previous studies have shown that automated malaria detection relies on the use of machine learning and deep learning algorithms. Given the robustness of DL algorithms in handling large-scale datasets of microscopic images, we adopted a DL-based framework for malaria detection using thin smear images. Among DL algorithms, VGG 16, ResNet50, and DenseNet-201 are dominant in disease diagnosis, such as malaria. Table 1 summarizes related works by publication year, dataset, results, pros, and cons.

## Background

This study was developed based on using ML and DL algorithms to diagnose malaria. The DL algorithms were employed to extract features of the utilized microscopic images, while the ML algorithms were used for classification into normal or abnormal images. In DL-based techniques, the CNN was the core of the network architecture with a modification in terms of the number of layers and the order of layers. In implementation, we adopted the ResNet50, the VGG 16, and DenseNet 201. Besides, the support vector machine (SVM), and Long Short-Term Memory (LSTM) networks were covered in Materials and Methods.

### ResNet 50

In 2016, the Residual Neural Network (ResNet), introduced by He et al., aims to enhance performance by incorporating residual connections between layers. These connections reduce losses, increase knowledge acquisition, improve training efficiency, and make the model more robust against overfitting. ResNet models consist of multiple layers, ranging from 34 to 1202, with variations in the number of residual blocks and fundamental operations. Among these, ResNet-50 is the most widely used version, comprising 49 convolutional layers and one fully connected (FC) layer^16^.

### The VGG16

In 2014, Oxford University scholars created the VGG16^15^. It consists of 13 convolutional layers divided into 5 segments, in addition to one segment that contains three fully connected layers^15,17^. Therefore, the basic architecture of the network, leading to its name, is “the VGG16”. Usually, each layer uses a 3 × 3 kernel filter to reduce the number of parameters and nonlinear effects. The VGG 16 is characterized by a deeper architecture that leads to a progressive learning feature from low level to high level. Its enhanced nonlinear expressive power enables it to capture more features and handle more complex input data efficiently^17^. These features make the VGG16 particularly useful for tasks such as image recognition and classification.

### DenseNet − 201

The DenseNet (Dense Convolutional Network) is a DL method that was designed to enhance propagation of features and reuse extracted features. It was originated by Huang et al. in 2017 [Ref. 17]. It connects each layer directly to every other layer in a feed-forward pattern. This configuration promotes information flow, which impacts the redundancy of parameters and minimizes the overfitting^18^. It resolves the issue of the vanishing gradient, allowing gradients to propagate more efficiently during backpropagation. Consequently, the DenseNet often achieves higher accuracy with a low number of parameters compared to other traditional DL models. This makes it more suitable for image classification and segmentation^19^. DenseNet-201’s densely connected layers enable it to capture intricate relationships between features, making it well-suited for tasks that demand fine-grained discrimination^20^.

## Materials and methods

**Fig. 1:**
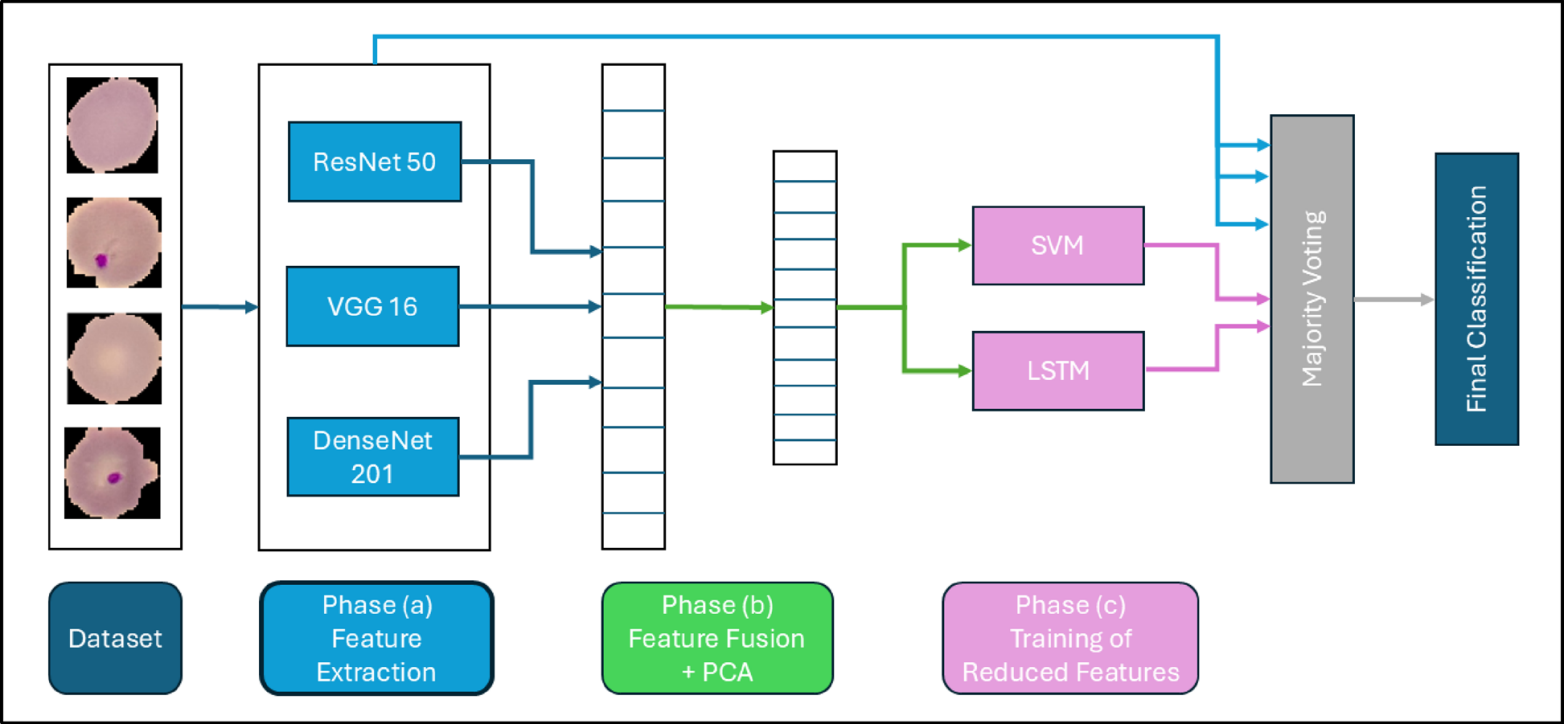
The proposed pipeline for malaria diagnosis illustrates **(a)** an End-to-End DL model, **(b)** adopted algorithms for feature extraction, and **(c)** adopted algorithms for classification.

The primary objective of this article is to develop and implement a robust AI-driven model for the accurate diagnosis of malaria using blood film microscopic images. The proposed methodology is designed as a multi-phase approach, with each phase contributing to the precision and reliability of the diagnostic process. The comprehensive workflow, as illustrated in Fig. 1, encompasses various stages of model development and implementation, including data preprocessing, feature extraction, model training, validation, and testing. This structured pipeline aims to ensure a high degree of diagnostic accuracy and efficiency, addressing the critical need for effective malaria detection in clinical and field settings.

1. Dataset.

The “Cell Images for Detecting Malaria” dataset^21^, hosted on Kaggle, is a well-curated collection of microscopic images of blood smears designed to facilitate research and development in malaria detection using machine learning and deep learning models. It comprises 27,558 images, equally divided into two categories: Parasitized (malaria-infected cells) and Uninfected (healthy cells). The images were captured using light microscopy at a consistent magnification of 100x, ensuring high-resolution visual clarity ideal for machine learning and deep learning applications. The original input image has a size of 150 × 150. The dataset was undergoing some preprocessing, such as resizing to be convenient with the input layer of the proposed AI model.

The dataset is systematically organized into two directories. The Parasitized directory contains images of red blood cells infected with the Plasmodium parasite, marked by distinct visual features such as irregular shapes, dark spots, and uneven internal textures. The Uninfected directory includes images of healthy red blood cells, characterized by their smooth texture, uniform shape, and absence of parasitic artifacts. This structure simplifies data loading and preprocessing tasks for researchers and developers.

One of the standout features of this dataset is its balanced distribution, with an equal number of images in each category. This balance minimizes class imbalance issues, ensuring that machine learning models can train effectively without bias toward a particular class. Additionally, the dataset exhibits biological diversity, encompassing a wide range of cell morphologies, staining variations, and patterns that mirror the complexities encountered in real-world clinical settings.

**Fig. 2:**
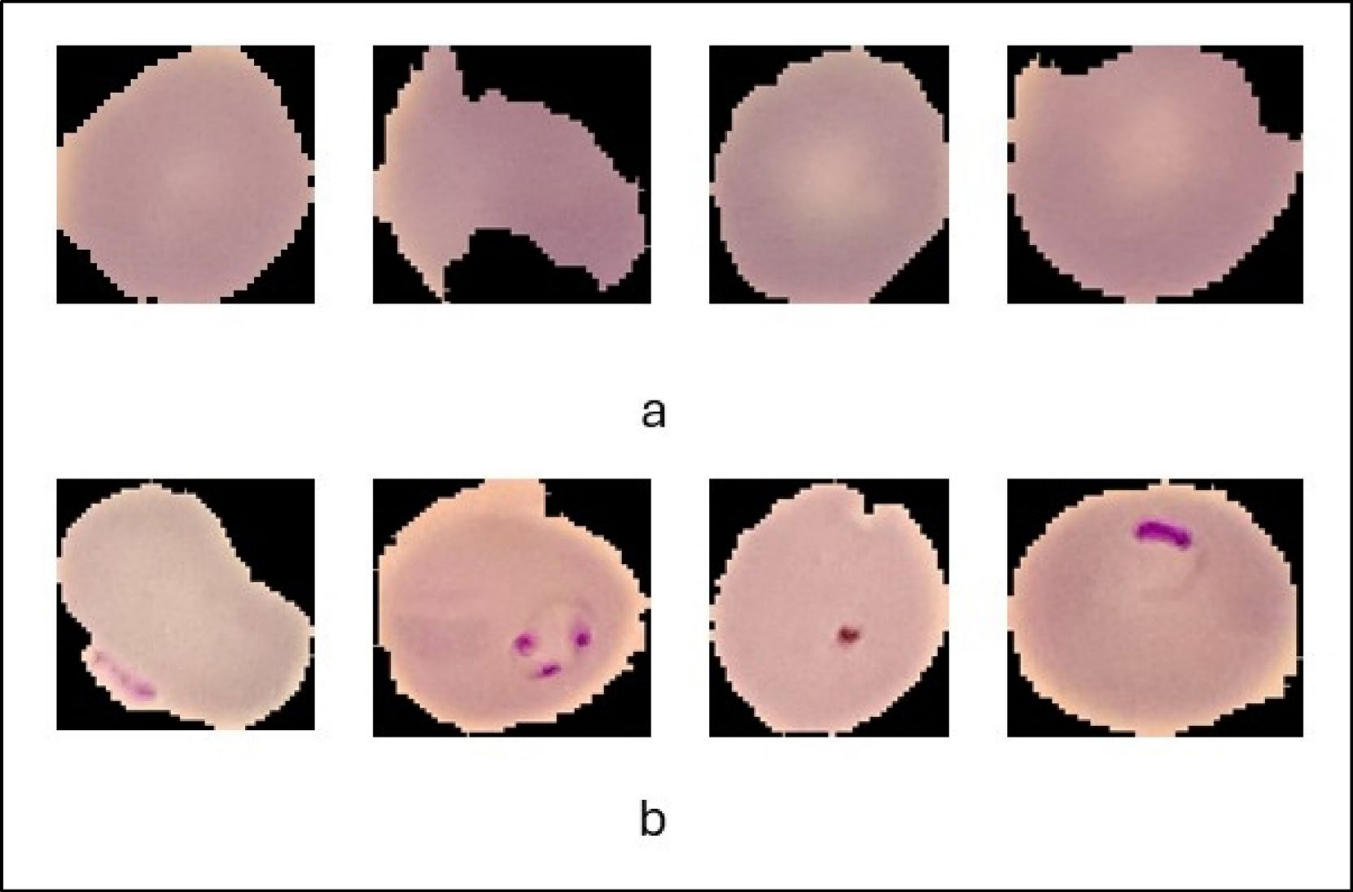
Samples for the utilized dataset: **(a)** uninfected samples, **(b)** infected samples.

Overall, the dataset is an invaluable resource for advancing AI-driven healthcare solutions. It is particularly suited for training, validating, and testing classification models as well as for transfer learning, where pre-trained models are fine-tuned for malaria detection tasks. The high-quality, diverse, and well-labelled nature of this dataset ensures that models trained on it are robust, reliable, and capable of performing effectively in real-world diagnostic scenarios. The dataset was split into 70% training, 15% validation, and 15% testing during the implementation of the proposed AI model. Examples of the utilized dataset are provided in Fig. 2. The distribution of the image count among the targeted classes is shown in Table 2.

**Table 1:** A comparison of related works concerning malaria diagnosis using AI techniques.

Study	Year	Dataset	Method	Results	Pros	Cons
Muhammad et al.^11^	2025	772 (616 + 156) images for rouleaux morphology and 772 for normal morphology	XceptionNet, ResNet 50, DenseNet, EfficientNetB4, customized CNN	Accuracy 99% for DeseNet-201	Rouleaux formation morphology of the RBCs is detected with DL algorithms.	Unbalance of Giemsa-stained images with field-stained images.
Ozsahin et al.^3^	2024	Related to 2207 patients	MLR, ANN, ANFIS, and RF	Model performance is evaluated in R, R^2^, RMSE, and MSE	Developing hybrid ML models for malaria diagnosis.	Using only ML algorithms. Generalization of the approach is limited.
Hoyos and Hoyos^6^	2024	222 original images and 666 augmented images	YOLOv8	An accuracy of 91% for original images and 95% for augmented images	Detecting malaria parasites and leukocytes with high accuracy.	Low diversity of trained images. Low number of used datasets.
Khan et al.^2^	2024	12,500 augmented microscopic images	R-CNN & ResNet- 18, 50, 101, 152 & GoogleNet	R-CNN outperformed the other classifiers with an accuracy of 91.21%	A novel framework was presented for malaria detection.	Cell morphological changes were not considered.
Hemachandran et al.^14^	2023	27,558 microscopic images	CNN & ResNet-50 & MobileNet-V2	MobileNet-V2 outperformed by an accuracy of 97%.	Comparing the performance of many DL models on a large dataset.	A traditional CNN was used.
Muhammad et al.^10^	2023	51 images (rouleaux morphology) and 180 images (normal morphology)	Customized CNN for normal morphology and rouleaux morphology	Accuracy: 90.95% (300 × 300) & 87.75% (500 × 500)	Evaluating the CNN performance in detecting abnormal cell morphology across varying image sizes.	A relatively low number of images (6088). Shallow depth of CNN layers (5 layers)
Ozsahin et al.^15^	2022	300 infected thick smear images and 319 thin smear images	Customized CNN, ResNet-50, VGG16, and Inception V3	Best performance of thick smear images with an accuracy of 96.97%.	Developing a novel model for malaria diagnosis based on DL techniques.	Relative law number of images.
Sunarko et al.^1^	2020	Related to 150 infected patients and 50 healthy patients (2000 images)	Otsu’s method + K -clustering	Accuracy: 94.6% Specificity:96.2% Sensitivity: 93%	A threshold-based segmentation method was adopted for malaria diagnosis.	Low amount of data. Detecting parasites in schizont stage is limited.
Kassim et al.^13^	2020	Polygen set was 34,226 RBC, and point set was 162,443 RBC	RBCNet based on U-Net and faster R-CNN	Cell detection with an accuracy of over 97%	A novel DL algorithm was presented for the RBCs segmentation using a large dataset.	No classification of infected and non-infected cells.

**Table 2:** Distribution of the dataset utilized.

Class	Uninfected	Infected
Training	9645	9645
Validation	2067	2067
Testing	2067	2067

2. Phase (a): Implementation of End-to-End Deep Learning Models.

In this phase, the methodology combines the power of transfer learning and end-to-end deep learning approaches to process microscopic blood film images for malaria diagnosis. Three state-of-the-art CNN architectures—ResNet-50^22^, VGG-16^23^, and DenseNet-201^24^—are employed as feature extractors. These models utilize pre-trained weights from large-scale datasets, enabling them to recognize intricate patterns in blood cell images. In these models, we performed transfer learning by retraining the end head of the CNN, including a fully connected layer, a dropout layer, a SoftMax layer, and a classification layer. Each of the proposed models keep its main parameters in the convolution layer including the number of filters, stride length, and its weights.

For ResNet-50, it captures 2048 deep hierarchical features through its residual connections, addressing vanishing gradient problems for effective learning. Meanwhile, VGG-16 identifies 4096 spatial features using its uniform convolutional architecture, ensuring consistent extraction of visual details. In addition, DenseNet-201 enhances feature propagation by creating densely connected layers, improving the richness and relevance of extracted 1920 features.

Beyond feature extraction, these architectures are also trained as end-to-end models. This allows the entire network—from input to classification layers—to be fine-tuned specifically for malaria diagnosis. By adapting pre-trained models to the task at hand, the networks learn to optimize both low-level and high-level features relevant to identifying infected and healthy cells. This dual approach not only harnesses the strengths of transfer learning but also capitalizes on the adaptability of deep learning models, creating a robust foundation for accurate malaria diagnosis.

In this phase, the methodology combines the strengths of transfer learning and end-to-end deep learning approaches to process microscopic blood film images for malaria diagnosis. Three advanced convolutional neural network (CNN) architectures—ResNet-50, VGG-16, and DenseNet-201—serve as the backbone for feature extraction and classification. These models leverage pre-trained weights from large-scale datasets like ImageNet, enabling them to detect intricate and subtle patterns in blood cell images with remarkable precision and efficiency. The process of feature extraction from CNN models can be mathematically represented as shown in Eq. (1).

1 $$\:F={f}_{CNN}\left(X;{\theta\:}_{pre-trained}\right)\:\:\:\:\:\:\:\:\:\:$$

Where $\:X\:$is the input image, $\:{f}_{CNN}$ represents the feature extraction function of the CNN model, $\:{\theta\:}_{pre-trained}$ Are the pretrained weights, $\:F$ Is the resulting feature vector.

Each architecture contributes uniquely to feature extraction. ResNet-50 captures 2048 deep hierarchical features through its residual learning framework, which introduces shortcut connections to bypass layers. This residual connection can be described as shown in Eq. (2).

2 $$\:y=f\left(x\right)+x$$

Where $\:x$ is the input for the residual block, $\:f\left(x\right)$ is the learned transformation, $\:y$ is the output of the residual block

VGG-16, known for its simple and sequential structure, extracts 4096 spatial features using uniform convolutional layers with small $\:3\times\:3$ Filters. Equation (3) presents the output of these layers.

3 $$\:{z}_{i,j,k}=\sum\limits_{m=0}^{M-1}\sum\limits_{n=0}^{N-1}{x}_{i+m,j+n}.\:{w}_{m,n,k}+{b}_{k}$$

Where $\:{z}_{i,j,k}$ is the output feature map, $\:{x}_{i+m,j+n}$ is the input feature map, $\:{w}_{m,n,k}$​ are the weights of the filter, $\:{b}_{k}\:$is the bias term, and $\:M$ and $\:N$ are the filter dimensions. This approach ensures precise detection of spatial characteristics such as cell morphology and texture.

DenseNet-201 enhances feature propagation and reuse through densely connected layers, where each layer is connected to all previous layers. This can be expressed as presented in Eq. (4).

4 $$\:{x}_{l}={H}_{l}\left(\right[{x}_{0},\:{x}_{1},\:\dots\:..,{x}_{l-1}\:\left]\right)$$

where $\:{x}_{l}$ is the output of the $\:{l}^{th}$ layer, $\:{H}_{l}$​ is the learned transformation, and $\:\left[{x}_{0},\:{x}_{1},\:\dots\:..,{x}_{l-1}\:\right]\:$represents the concatenation of feature maps from preceding layers. This connectivity results in the extraction of 1920 highly relevant features, contributing to a rich feature set.

Beyond feature extraction, these CNN architectures are fine-tuned as end-to-end deep learning models. Fine-tuning optimizes the parameters. $\:{\theta\:}_{fine-tuned}$ for the specific task of malaria diagnosis by minimizing a loss function as shown in Eq. (5).

5 $$\:{\theta\:}_{fine-tuned}=\text{arg}\text{min}L(f\left(X;\theta\:\right),y)$$

where $\:f\left(X;\theta\:\right)$ is the predicted output of the model, $\:y$ is the ground truth label, and $\:L$ is the cross-entropy loss function. This process ensures that the models learn both low-level features (such as textures and shapes) and high-level features (such as parasitic patterns) unique to blood smear microscopy.

**Fig. 3:**
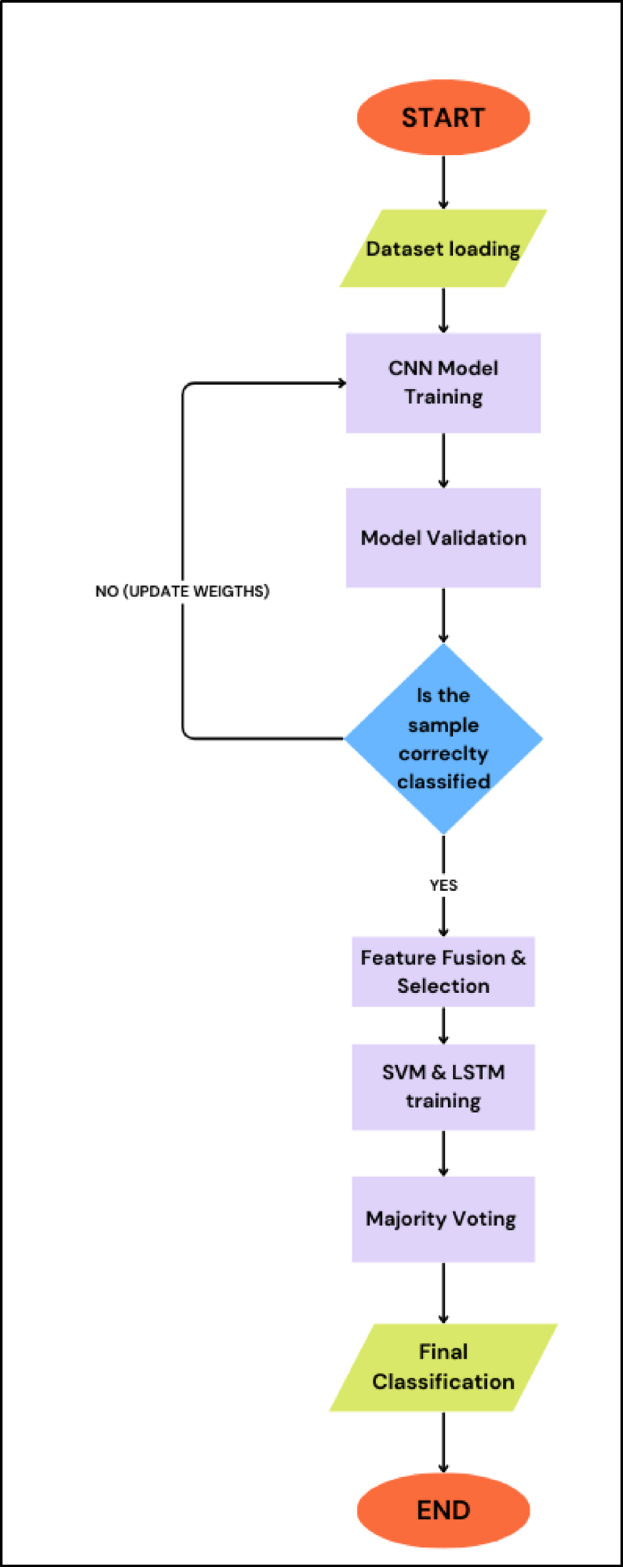
Flow chart of the proposed approach for malaria detection.

For more information, Table 3 summarizes the training hyperparameters used for the training of the proposed models, reflecting a carefully tuned configuration that balances learning stability, efficiency, and generalization performance through mini-batch optimization, learning rate scheduling, and periodic validation. As the selection of training hyperparameters may directly influence the proposed models^25^. Moreover, Fig. 3 provides a flow chart of the proposed algorithm, including the training and testing phases.

**Table 3:** Different training hyperparameters for the proposed CNN models.

Hyperparameter	Value	Description
Optimizer	SGDM	Stochastic Gradient Descent with Momentum
Execution Environment	Parallel	Utilizes multiple CPU/GPU cores for faster computation
Mini-Batch Size	64	Number of samples processed per training iteration
Maximum Epochs	30	Number of complete passes through the training dataset
Activation Function	ReLU	It introduces non-linearity and helps accelerate convergence by allowing models to learn complex patterns efficiently.
Initial Learning Rate	1.00E-05	Starting value for learning rate
Learning Rate Schedule	piecewise	Learning rate is adjusted at pre-defined intervals
Validation Frequency	50	Validation is performed every 50 iterations

3. Phase (b): Feature Fusion and dimensionality reduction.

Following the independent extraction of features using ResNet-50, VGG-16, and DenseNet-201, the next step in the methodology involves feature fusion. This process combines the diverse feature sets generated by these models into a unified, comprehensive feature vector. The fusion operation is performed through concatenation, which aggregates the feature vectors from the three models according to Eq. (6).

6 $$\:{F}_{fused}=[{F}_{ResNet}\:,{F}_{VGG\:16}\:,\:{F}_{DenseNet}]$$

Where $\:{F}_{ResNet}\:,{F}_{VGG\:16}\:,\:{F}_{DenseNet}$ are the feature vectors extracted from ResNet-50, VGG-16, and DenseNet-201, respectively, $\:{F}_{fused}$ Represents the fused feature vector. The total number of features after concatenation is 8064.

This fused feature vector provides a richer representation by combining both global patterns (captured by ResNet-50) and local spatial details (captured by VGG-16 and DenseNet-201). While the combined features significantly enhance the model’s capability to distinguish between malaria-infected and healthy cells, their high dimensionality poses challenges in terms of computational complexity and overfitting.

To address these challenges, Principal Component Analysis (PCA) is employed for dimensionality reduction. PCA transforms the high-dimensional fused feature vector into a lower-dimensional space by finding the principal components that capture the maximum variance in the data. The transformation can be represented as shown in Eq. (7).

7 $$\:{F}_{PCA}=\:{F}_{fused}\:W$$

Where $\:{F}_{fused}$ is the original fused feature matrix of size $\:N\times\:8064,\:$*N* is the number of samples. The projection matrix *W* is computed by solving the eigenvalue decomposition of the covariance matrix of $\:{F}_{fused}$.

8 $$\:C=\frac{1}{N}{f}_{fused}^{T}{F}_{fused}$$

Equation 8 introduces the covariance matrix (C) of the fused feature matrix​. It is a square matrix that captures the relationships between the features in the fused feature space. By selecting only the top $\:k$ Principal components that capture 95% of the variance, PCA ensures that the most discriminative features are preserved while reducing the feature space. This reduction minimizes computational complexity, mitigates overfitting, and accelerates subsequent classification tasks, all while maintaining high diagnostic accuracy.

4. Phase (c): Hybrid Classification Framework.

After dimensionality reduction, the refined feature vector is processed through a hybrid classification framework that combines the strengths of traditional machine learning and deep learning models. This framework consists of two components: Support Vector Machine (SVM) and Long Short-Term Memory Networks (LSTM). Each component plays a vital role in ensuring robust and accurate classification, complementing each other’s capabilities.

- Support Vector Machine (SVM).

SVM is a powerful supervised learning algorithm that excels in identifying optimal decision boundaries between classes, even in high-dimensional spaces^26^. In this framework, the reduced feature vector is provided as input to the SVM classifier, which works by finding a hyperplane that maximally separates the two classes: malaria-infected and healthy samples. The SVM classifier aims to maximize the margin, which is the distance between the hyperplane and the nearest data points on either side, known as support vectors. This approach ensures that the classifier generalizes well to unseen data, making it particularly effective for binary classification tasks like malaria diagnosis. The robustness and reliability of SVM make it an essential component of this hybrid framework. The core objective of SVM is to maximize the margin between the separating hyperplane and the closest data points from each class, known as the support vectors. The optimal hyperplane can be mathematically expressed in Eqs. (9–11)

9 $$\:f\left(x\right)={w}^{T}x+b=0$$

Where $\:x$ is the input feature vector, $\:w$ is the weight vector orthogonal to the hyperplane, $\:b$ is the bias term.

To find the optimal hyperplane, SVM solves the following convex optimization problem:

10 $$\:\underset{a,{\:b}}{\text{min}}\frac{1}{2}{\parallel w\parallel}^{2}\:\:\:\:$$

11 $$\:subject\:to:\:{y}_{i}\left({w}^{T}{x}_{i}+b\right)\ge\:1,\:\:\:{\forall\:}_{i}$$

Where $\:{y}_{i}\in\:\left\{1,-1\right\}$ *are the class labels*, $\:{x}_{i}$ *are the training instances*.

By maximizing the margin $\:\frac{2}{\parallel w\parallel}$, SVM minimizes overfitting and improves generalization performance on unseen test data. For non-linearly separable data, SVM incorporates kernel functions $\:K({x}_{i},\:{x}_{j})$to map data into a higher-dimensional space, where linear separation becomes feasible. In the context of this study, the SVM classifier processes the transformed PCA feature set to learn the best separating hyperplane between infected and uninfected cells. Its mathematical rigor, geometric interpretation, and generalization capability make SVM an indispensable component of the proposed hybrid classification system.

b. Long Short-Term Memory Networks (LSTM).

LSTM, a type of recurrent neural network (RNN), is used in this framework to capture patterns and contextual relationships in the data. Although the feature vector itself is not sequential, LSTM networks excel at modelling dependencies among features, allowing the system to recognize intricate patterns indicative of malaria-infected cells^12,19^. LSTMs are designed with specialized mechanisms, such as gates that regulate the flow of information, ensuring the model retains only the most relevant patterns and discards unnecessary details. This makes LSTMs particularly adept at learning complex and nuanced representations from data, adding an extra layer of interpretability and robustness to the classification process. In the context of malaria diagnosis, LSTM adds an interpretive layer capable of capturing complex feature interactions that may not be linearly separable^27^.

LSTM achieves this through a gated memory cell architecture, which consists of the forget gate, input gate, cell state update, and output gate. These gates control the flow of information, selectively remembering or forgetting parts of the input and prior hidden states, thereby enabling the network to focus on the most informative patterns while discarding irrelevant ones. The internal operations of an LSTM cell at time step $\:t$ are described by the following Eqs. (12–17):

- Forgot gate.

12 $$\:{f}_{t}=\sigma\:\left({W}_{f}\cdot\:\left[{h}_{t-1},{x}_{t}\right]+{b}_{f}\right)$$

- Input gate.
  13 $$\:{i}_{t}=\sigma\:\left({W}_{i}\cdot\:\left[{h}_{t-1},{x}_{t}\right]+{b}_{i}\right)\:\:\:\:$$

14 $$\:{\stackrel{\sim}{C}}_{t}=tanh\left({W}_{c}\cdot\:\left[{h}_{t-1},{x}_{t}\right]+{b}_{c}\right)\:\:$$

- Cell state update.
  15 $$\:{C}_{t}={f}_{t}\cdot\:{C}_{t-1}+\:{i}_{t}\cdot\:\:{\stackrel{\sim}{C}}_{t-1}\:\:\:$$

- Output gate.
  16 $$\:{o}_{t}=\sigma\:\left({W}_{o}\cdot\:\left[{h}_{t-1},{x}_{t}\right]+{b}_{o}\right)\:\:$$

17 $$\:{h}_{t}=\:{o}_{t}\cdot\:\text{tanh}\left({C}_{t}\right)\:\:$$

Where $\:{x}_{t}\:$is the input at time step $\:t$, $\:{h}_{t-1}$ is the previous hidden state, $\:{C}_{t}$ is the current cell state, $\:\sigma\:$ is the sigmoid activation function, $\:W\:and\:b$ are the trainable weights and biases, $\:tanh$ is the hyperbolic tangent activation. These mechanisms enable LSTM to retain long-term dependencies and filter out noise, which is critical when learning from complex biological patterns such as variations in red blood cell morphology in malaria.

The designed RNN architecture incorporates an LSTM-based deep learning structure tailored for classifying malaria-infected versus uninfected samples using the reduced feature set obtained from PCA. The network begins with a sequence input layer configured for an input dimension of 3135 features, representing the principal components derived from the original high-dimensional fused feature vector. This is followed by an LSTM layer with 128 hidden units, allowing the network to capture temporal or contextual dependencies across the input features, even though they are not time-series data in the classical sense.

**Fig. 4:**
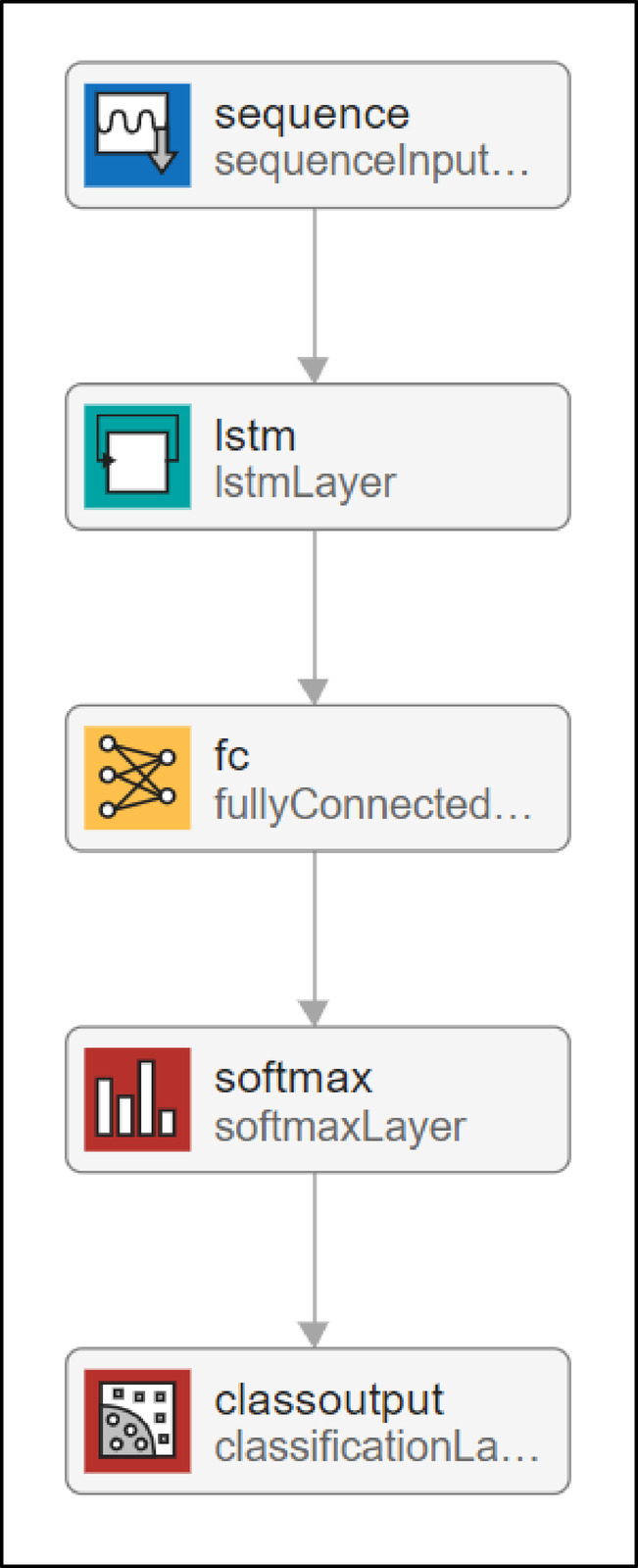
Structure of the proposed RNN (LSTM) model for malaria detection.

The LSTM layer is succeeded by a fully connected layer that maps the learned feature representations to the desired number of output classes (in this case, two). A SoftMax layer follows, which converts the raw class scores into normalized probabilities, enabling probabilistic interpretation of the classification outputs. Finally, the classification layer computes the loss during training and evaluates the model’s prediction performance. This architecture effectively leverages the LSTM’s capacity to model complex inter-feature relationships, improving the system’s ability to differentiate between malaria-infected and uninfected blood samples. Figure 4 depicts the detailed structure of the proposed network.

5. Decision Aggregation.

To further improve the reliability and accuracy of the classification process, the methodology incorporates a majority voting mechanism. This mechanism is used to aggregate the predictions from multiple classifiers, including the three end-to-end deep learning models (ResNet-50, VGG-16, and DenseNet-201), as well as the SVM and LSTM classifiers. By combining the outputs of these diverse models, majority voting ensures a consensus-based decision, reducing the likelihood of errors arising from the limitations of any single model^28^.

In the majority voting process, each classifier independently predicts whether the input blood film image corresponds to a malaria-infected or non-infected sample. These predictions are treated as votes, with each model contributing one vote to the final decision. The class label with the majority of votes is selected as the final classification outcome. For instance, if the five models produce predictions as follows:

- ResNet-50: Malaria-infected.
- VGG-16: Malaria-infected.
- DenseNet-201: Non-infected.
- SVM: Malaria-infected.
- LSTM: Non-infected.

Hence, the majority class (Malaria-infected in this case) is chosen as the final decision.

This approach is particularly effective in leveraging the complementary strengths of the models involved. The deep learning models provide robust feature extraction and pattern recognition capabilities, while the SVM contributes precise decision boundary optimization, and the LSTM captures complex relationships within the data. By aggregating these diverse perspectives, majority voting minimizes the influence of outlier predictions and improves overall classification robustness.

Moreover, majority voting is inherently adaptable and scalable. Additional classifiers can be integrated into the voting process without significant alterations to the system, further enhancing its versatility. This mechanism also reduces the impact of noisy data or model-specific biases, as the final decision reflects a consensus rather than relying on a single classifier’s output. The outcome of the majority voting process provides the final classification, reliably determining whether the input blood film image is malaria-infected or non-infected. This consensus-based strategy ensures that the diagnostic system delivers high levels of accuracy and confidence, making it suitable for real-world clinical applications where reliability is critical.

## Results

**Fig. 5:**
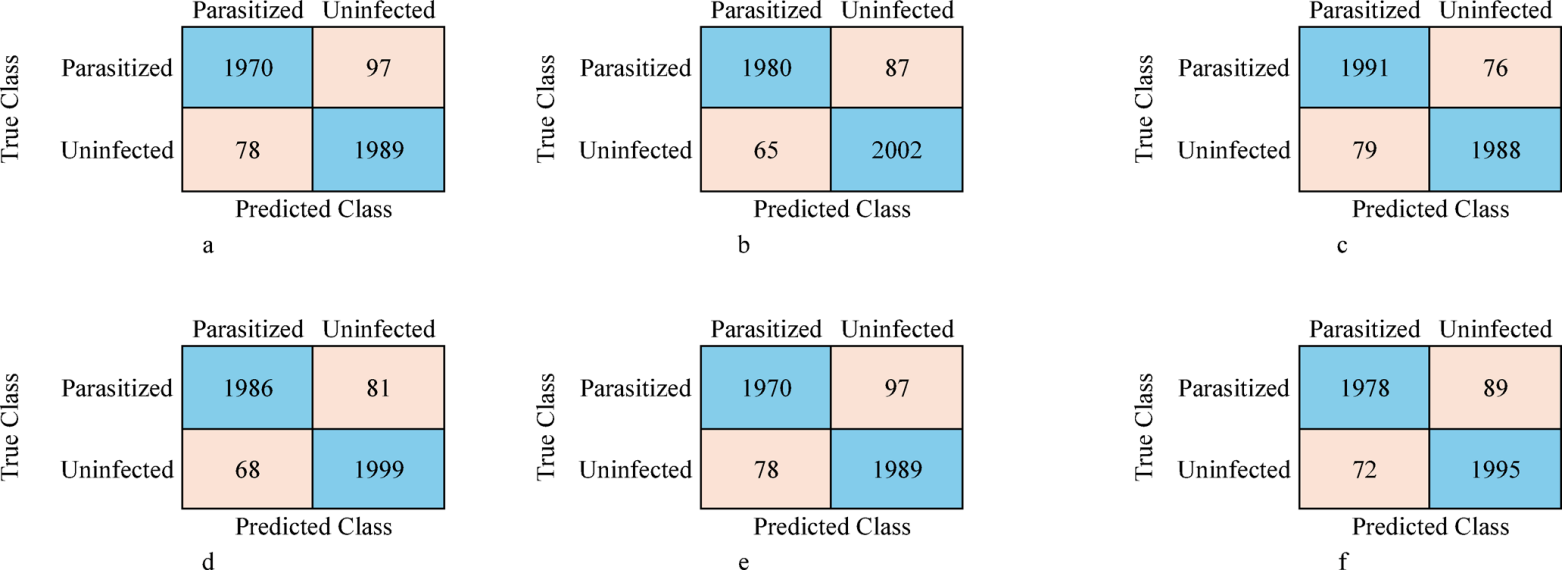
Confusion matrices for the proposed models: **(a)** ResNet-50, **(b)** VGG-16, **(c)** DenseNet-201, **(d)** SVM, **(e)** LSTM, **(f)** Majority voting.

The main objective of the study was to implement an automated framework to accurately classify the blood smear image for the diagnosis of Malaria. The performance of the proposed malaria detection methodology was evaluated across different models, including ResNet-50, VGG-16, DenseNet-201, Support Vector Machine (SVM), Long Short-Term Memory (LSTM) networks, and Majority Voting. The evaluation was conducted using multiple metrics, including Accuracy, Sensitivity (SEN), Specificity (SPE), Precision (PRE), Error Rate, False Positive Rate (FPR), False Negative Rate (FNR), Negative Predictive Value (NPV), F1-Score, and Matthews Correlation Coefficient (MCC) using the following equations^12^ in terms of TP: True Positive, TN: True Negative, FP: False Positive, and FN: False Negative using the obtained confusion matrices in Fig. 5.

18 $$\:\text{A}\text{c}\text{c}\text{u}\text{r}\text{a}\text{c}\text{y}\:=\:(\text{T}\text{P}\hspace{0.17em}+\hspace{0.17em}\text{T}\text{N})\:/\:(\text{T}\text{P}\hspace{0.17em}+\hspace{0.17em}\text{F}\text{N}\hspace{0.17em}+\hspace{0.17em}\text{F}\text{P}\hspace{0.17em}+\hspace{0.17em}\text{T}\text{N})$$

19 $$\:\text{S}\text{e}\text{n}\text{s}\text{i}\text{t}\text{i}\text{v}\text{i}\text{t}\text{y}\hspace{0.17em}=\hspace{0.17em}\text{T}\text{P}\:/\:(\text{T}\text{P}\hspace{0.17em}+\hspace{0.17em}\text{F}\text{N})$$

20 $$\:\text{E}\text{r}\text{r}\text{o}\text{r}\:\text{R}\text{a}\text{t}\text{e}\:=\:(\text{F}\text{P}\hspace{0.17em}+\hspace{0.17em}\text{F}\text{N})\:/\:(\text{T}\text{P}\hspace{0.17em}+\hspace{0.17em}\text{F}\text{N}\hspace{0.17em}+\hspace{0.17em}\text{F}\text{P}\hspace{0.17em}+\hspace{0.17em}\text{T}\text{N})$$

21 $$\:\text{Sp}\text{e}\text{c}\text{i}\text{f}\text{i}\text{c}\text{i}\text{t}\text{y}\hspace{0.17em}=\hspace{0.17em}\text{T}\text{N}\:/\:(\text{T}\text{N}\hspace{0.17em}+\hspace{0.17em}\text{F}\text{P})$$

22 $$\:\text{P}\text{r}\text{e}\text{c}\text{i}\text{s}\text{i}\text{o}\text{n}\hspace{0.17em}=\hspace{0.17em}\text{T}\text{P}\:/\:(\text{T}\text{P}\hspace{0.17em}+\hspace{0.17em}\text{F}\text{P})$$

23 $$\:\text{F}\text{a}\text{l}\text{s}\text{e}\:\text{P}\text{o}\text{s}\text{i}\text{t}\text{i}\text{v}\text{e}\:\text{R}\text{a}\text{t}\text{e}\hspace{0.17em}=\hspace{0.17em}\text{F}\text{P}\:/\:(\text{F}\text{P}\hspace{0.17em}+\hspace{0.17em}\text{T}\text{N})$$

24 $$\:\text{F}\text{a}\text{l}\text{s}\text{e}\:\text{N}\text{e}\text{g}\text{a}\text{t}\text{i}\text{v}\text{e}\:\text{R}\text{a}\text{t}\text{e}\hspace{0.17em}=\hspace{0.17em}\text{F}\text{N}\:/\:(\text{F}\text{N}\hspace{0.17em}+\hspace{0.17em}\text{T}\text{P})$$

25 $$\:\text{N}\text{e}\text{g}\text{a}\text{t}\text{i}\text{v}\text{e}\:\text{P}\text{r}\text{e}\text{d}\text{i}\text{c}\text{t}\text{i}\text{v}\text{e}\:\text{V}\text{a}\text{l}\text{u}\text{e}\hspace{0.17em}=\hspace{0.17em}\text{T}\text{N}\:/\:(\text{T}\text{N}\hspace{0.17em}+\hspace{0.17em}\text{F}\text{N})$$

26 $$\:\text{F}1-\text{S}\text{c}\text{o}\text{r}\text{e}\:=\text{F}1-\text{S}\text{c}\text{o}\text{r}\text{e}\:=\:(2\:\times\:\:(\text{S}\text{e}\text{n}\text{s}\text{i}\text{t}\text{i}\text{v}\text{i}\text{t}\text{y}\:\times\:\:\text{P}\text{r}\text{e}\text{c}\text{i}\text{s}\text{i}\text{o}\text{n}\left)\right)/\left(\text{S}\text{e}\text{n}\text{s}\text{i}\text{t}\text{i}\text{v}\text{i}\text{t}\text{y}\hspace{0.17em}+\hspace{0.17em}\text{P}\text{r}\text{e}\text{c}\text{i}\text{s}\text{i}\text{o}\text{n}\right)$$

27 $$\:\text{M}\text{C}\text{C}\:=\:\:(\text{T}\text{P}\times\:\text{T}\text{N}\:-\:\text{F}\text{P}\times\:\text{F}\text{N})\:/\sqrt{(\text{T}\text{P}+\text{F}\text{P})(\text{T}\text{P}+\text{F}\text{N})(\text{T}\text{N}+\text{F}\text{P})(\text{T}\text{N}+\text{F}\text{N})}\:$$

**Fig. 6:**
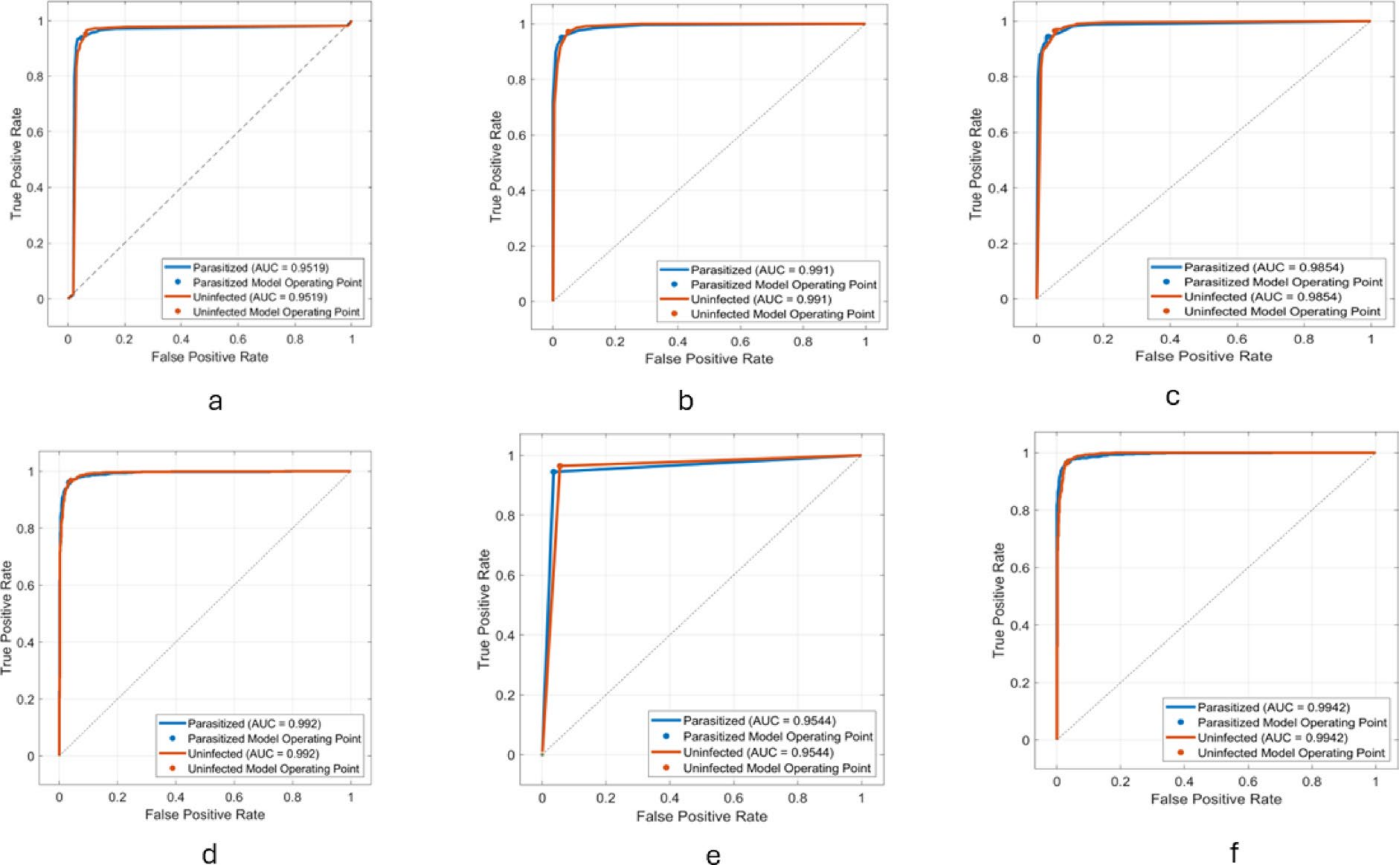
ROC curves for the proposed models: **(a)** ResNet-50, **(b)** VGG-16, **(c)** DenseNet-201, **(d)** SVM, **(e)** LSTM, and **(f)** Majority voting.

In the context of the current study on malaria classification using microscopic blood smear images, the cases in the confusion matrix are interpreted as follows, based on the two main classes: ‘Parasitized’ (malaria-infected) and ‘Uninfected’ (non-malaria), as mentioned in Table 4. Receiver Operating Characteristic (ROC) curves for the proposed models are shown in Fig. 6.

**Table 4:** Description of the targeted classes of malaria detection.

Case	Description
**True Positive (TP)**	The model correctly predicts a sample as **‘Parasitized’**, and it is actually **‘Parasitized’**.
**True Negative (TN)**	The model correctly predicts a sample as **‘Uninfected’**, and it is actually **‘Uninfected’**.
**False Positive (FP)**	The model incorrectly predicts a sample as **‘Parasitized’**, but it is actually **‘Uninfected’**.
**False Negative (FN)**	The model incorrectly predicts a sample as **‘Uninfected’**, but it is actually **‘Parasitized’**.

- Performance of transfer learning models.

**Fig. 7:**
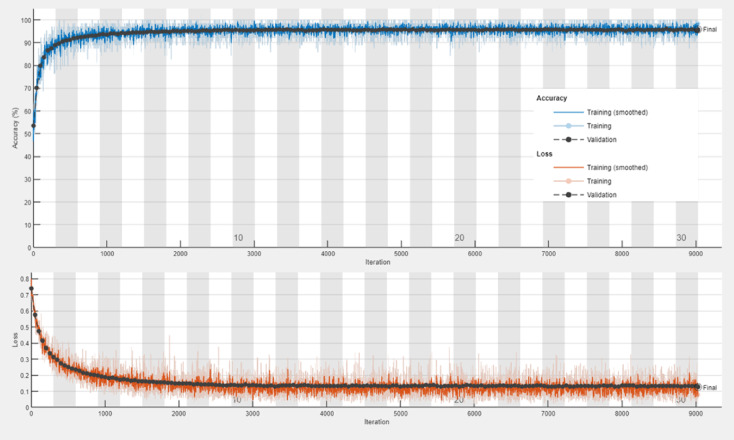
Training progress curve for the ResNet-50 model.

**Fig. 8:**
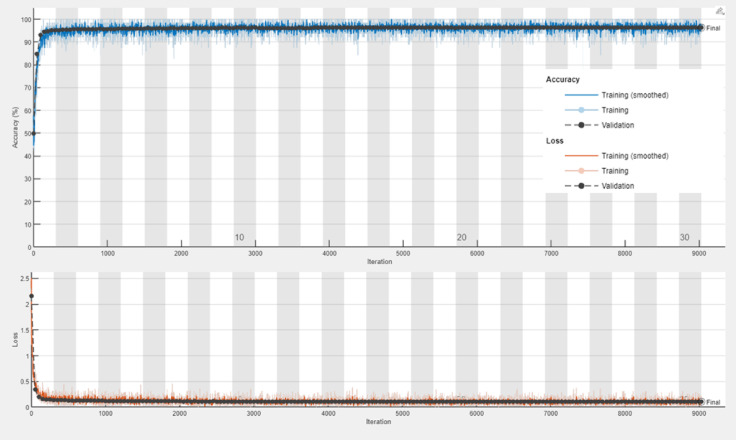
Training progress curve for the VGG-16 model.

**Fig. 9:**
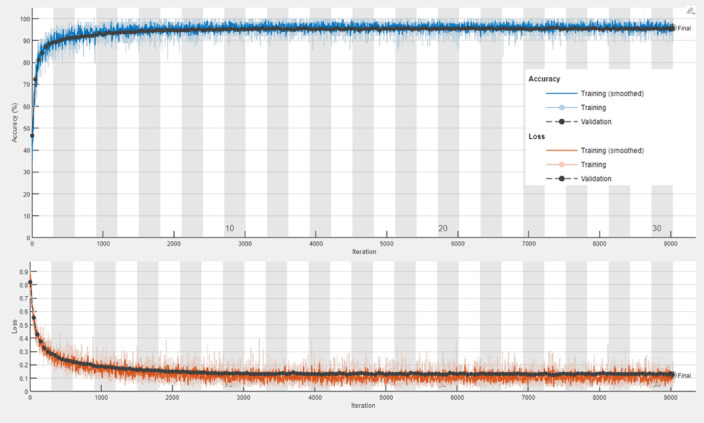
Training progress curve for DenseNet-201 model.

The transfer learning models, ResNet-50, VGG-16, and DenseNet-201, demonstrated robust performance in malaria detection, leveraging their pre-trained architectures for feature extraction and classification. ResNet-50 achieved an accuracy of 95.77%, with a sensitivity of 95.31% and specificity of 96.23%, effectively capturing hierarchical patterns in the data. VGG-16 obtained an accuracy of 96.32%, showing a well-balanced performance with sensitivity (95.79%) and specificity (96.86%). DenseNet-201 achieved an accuracy of 96.25% and the highest sensitivity (96.32%), indicating its exceptional ability to detect malaria-infected samples. All three models delivered competitive results in terms of F1-Score (ranging from 95.75 to 96.30%) and Matthews Correlation Coefficient (MCC) (91.96–93.15%), showcasing their reliability and effectiveness in automated malaria diagnosis. For monitoring the performance of the proposed models, training progress curves including training accuracy, validation accuracy, training loss, and validation loss has been recorded in Figs. 7, 8 and 9 for ResNet-50, VGG-16, and DenseNet-201, respectively.

b. PCA Results for Dimensionality Reduction.

**Fig. 10:**
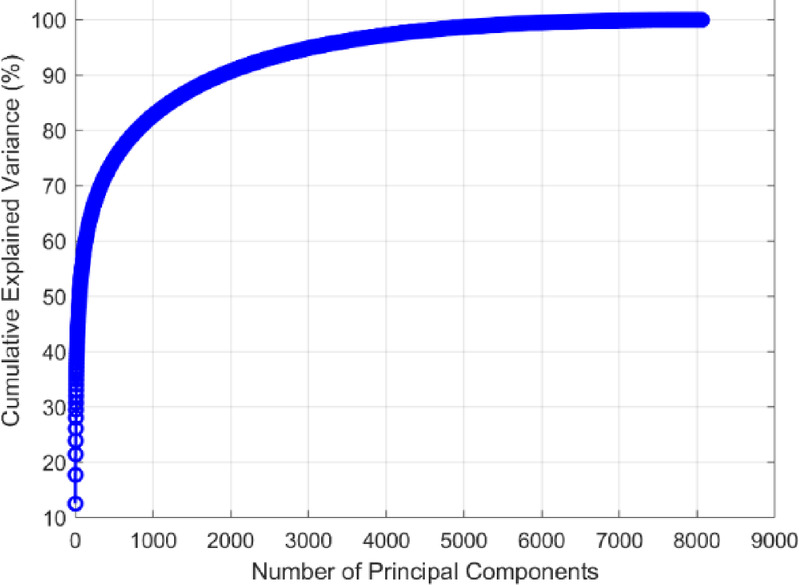
PCA Cumulative explained variance curve for malaria diagnosis features reduction.

PCA was applied to the high-dimensional fused feature set of 8064 features to reduce its dimensionality while preserving the most significant information. As depicted in Fig. 10, the cumulative explained variance is plotted against the number of principal components. The curve shows a steep initial rise, indicating that a substantial proportion of the variance in the dataset is captured by the first few principal components.

From the graph, it can be observed that the explained variance plateaus after a certain number of components, with diminishing returns from including additional components. Based on the analysis, 3135 principal components were selected, ensuring that the majority of the variance in the original data is retained. This reduction represents a significant compression of the feature space, approximately 61.2% of the original size, without a substantial loss of critical information.

c. Performance of the SVM model.

SVM achieved an accuracy of 96.40%. It showed strong specificity (96.71%) and sensitivity (96.08%), which indicated its capability to distinguish between infected and uninfected samples effectively. The F1-Score (96.38%) and MCC (93.09%) also indicated excellent balance and robustness.

d. Performance of the LSTM model.

LSTM achieved an accuracy of 96.11% and, sensitivity of 95.69%, which indicated that LSTM might miss some infected samples. However, it still showed a strong specificity of 96.52%, making it effective at identifying uninfected samples. The precision (96.49%) and error rate (3.89%) were similar to those of ResNet-50, indicating that LSTM performed well, but with a slight trade-off in sensitivity.

e. Overall majority voting performance.

The Majority Voting method, which integrates the predictions from ResNet-50, VGG-16, DenseNet-201, SVM, and LSTM, achieved the highest accuracy of 96.47%, outperforming all individual models. This approach also achieved the highest specificity (96.90%) and precision (96.88%), indicating that it was the most reliable method for identifying both malaria-infected and uninfected samples. The sensitivity (96.03%) and F1-Score (96.45%) were also high, contributing to the overall strength of the Majority Voting model. The detailed results for the proposed models are presented in the Table 5.

To evaluate the individual and collective impact of the core components in the proposed framework, an ablation study was conducted using ResNet-50, VGG-16, DenseNet-201, SVM, LSTM, and the final majority voting ensemble, as shown in Table 6. The results demonstrate the standalone effectiveness of each deep learning model, with VGG-16 achieving the highest individual accuracy (96.32%) among the CNNs. When the fused and PCA-reduced features were classified independently using SVM and LSTM, the performance either matched or exceeded that of the CNNs, indicating that the hybrid classifiers effectively capture discriminative patterns not fully exploited by the end-to-end deep networks. The final ensemble, integrating outputs from all models through a majority voting mechanism, yielded the highest performance across all metrics, with an accuracy of 96.47%, an F1-score of 96.45%, and a Matthews Correlation Coefficient of 93.35%. These results validate that each module—CNN feature extraction, SVM-LSTM hybrid classification, and majority voting—contributes meaningfully to the overall system, with the ensemble leveraging their complementary strengths to achieve optimal diagnostic accuracy.

**Table 5:** Detailed results for the proposed models for malaria diagnosis.

Criteria	ResNet 50	VGG 16	DenseNet 201	PCA + SVM	PCA + LSTM	Majority Voting
Accuracy	95.77%	96.32%	96.25%	96.40%	96.11%	**96.47%**
Sensitivity	95.31%	95.79%	**96.32%**	96.08%	95.69%	96.03%
Specificity	96.23%	96.86%	96.18%	96.71%	96.52%	**96.90%**
Precision	96.19%	96.82%	96.18%	96.69%	96.49%	**96.88%**
Error Rate	4.23%	3.68%	3.75%	3.60%	3.89%	**3.53%**
False Positive Rate	3.77%	3.14%	3.82%	3.29%	3.48%	**3.10%**
False Negative Rate	4.69%	4.21%	**3.68%**	3.92%	4.31%	3.97%
Negative Predictive Value	95.35%	95.84%	**96.32%**	96.11%	95.73%	96.07%
F1-Score	95.75%	96.30%	96.25%	96.38%	96.09%	**96.45%**
Matthews Correlation Coefficient	91.96%	93.15%	92.43%	93.09%	92.60%	**93.35%**

**Table 6:** Ablation study showing the individual and combined performance of CNN-based classifiers.

Model	Accuracy	F1-Score	MCC	Key Observation
CNN backbones only • ResNet-50 • VGG-16 • DenseNet-201	95.77–96.32%	95.75–96.30%	91.96–93.15%	Establishes a strong deep-learning baseline. DenseNet-201 gives the best recall (SEN = 96.32%).
Hybrid classical models only • SVM • LSTM	96.40% − 96.11%	96.38% − 96.09%	93.09% − 92.60%	Shows that the PCA-based feature-fusion vector, when fed to SVM or LSTM, already surpasses or matches the best single CNN.
Ensemble layer • Majority Voting (CNN + SVM + LSTM)	96.47%	96.45%	93.35%	Delivers the global optimum across every metric

## Discussion

This study presents an experimental setting to detect malaria by examining a set of thin smear blood images using a combination of AI-based techniques. As an end-to-end DL model, the transfer learning methodology exemplified by ResNet-50, VGG 16, and DenseNet-201 was employed. We investigated both the SVM and LSTM models as ML algorithms. Moreover, we proposed majority voting as an aggregate decision for malaria detection. Each technique was evaluated through the given evaluation metrics as described in Results. In this study, we employed three widely recognized convolutional neural network architectures—ResNet-50, VGG-16, and DenseNet-201—as feature extractors and end-to-end classifiers. These models remain highly relevant and effective in the context of medical image analysis due to their proven robustness, interpretability, and transfer learning capabilities. VGG-16 offers a straightforward and uniform architecture that simplifies model interpretation and tuning. ResNet-50 introduces residual connections, which effectively address vanishing gradient problems and enable the training of deeper, more expressive networks. DenseNet-201 leverages dense connectivity to enhance feature reuse and gradient flow, which is particularly beneficial when working with fine-grained features in microscopic blood smear images. These architectures have been extensively validated in numerous biomedical imaging studies, providing a strong foundation for benchmarking and performance comparison. While newer models such as ConvNeXt^29^, EfficientNet-V2, Vision Transformers (ViT)^30,31^, and MobileNet V3 offer promising results on large-scale datasets, they often require more complex tuning, higher computational resources, and substantial training data—conditions that are not always ideal in medical imaging contexts. Therefore, the selected architectures strike a practical balance between accuracy, efficiency, and generalizability, making them well-suited for the task of automated malaria diagnosis^32,33^.

Notably, Table 5 shows that the voting majority technique outperformed the other techniques in terms of accuracy, specificity, precision, error rate, false positive rate, F1-Score, and Mattews correlation coefficient. Obviously, the DenseNet-201 has recorded the best results in sensitivity, false negative rate, and negative predictive value. Using PCA to optimize the used features has proven its ability to enhance the results, demonstrating significance in detection with less computing time. In the realm of AI, the majority voting techniques permit a collaboration between all involved algorithms to vote according to the dominant decision. This entails taking advantage of a transition from traditional AI algorithms to leveraging the use of groups in decision-making. This fact is proven through our results as illustrated in Table 5. The DenseNet-201 performed efficiently compared to the other algorithms because of its ability to reuse features map through the dense layers leading to minimizing overfitting and vanishing gradients. Furthermore, as noted in training and losses performance curves, DenseNet-201 had the best one.

Notably, a comparison with the studies in Table 1 reveals that none have proposed the use of majority voting for malaria detection. While many existing studies are hampered by limited datasets, this research builds upon the foundation laid by earlier works, particularly those referenced as^2,13^, which demonstrate the potential of larger data pools. Furthermore, our study utilized the same dataset as^13^. However, we expanded the analysis by incorporating additional algorithms alongside the majority voting technique, achieving approximately the same level of accuracy.

The study’s limitations include its failure to account for morphological changes in the examined cells. Additionally, different datasets should be used to validate the majority voting method. To show how well the suggested methodology performs over them, additional thick smear blood images should be evaluated.

## Conclusions

The study presented an AI-based multi-model framework for malaria detection. In this context, the majority voting represents a significant advancement, setting this study apart from previous research that has not ventured into this innovative way. This approach signifies a meaningful advancement in the field of medical image analysis, as it enhances the robustness and reliability of diagnostic outcomes. Therefore, this ensemble strategy reduces the likelihood of misclassification and strengthens the overall decision-making process.

A key contribution of this study lies in the utilization of a large and diverse dataset, which plays a critical role in improving the generalization capabilities of the model. In other words, a large dataset pool not only improves the results but also paves the way for further methods to detect malaria. Moreover, using thin smear images provides reliable results, demonstrating that malaria detection is unaffected by the microscopic images utilized. This finding confirms that the effectiveness of the detection process remains constant, regardless of variations in microscopic images’ characteristics. Ultimately, this study highlights the importance of evolving methodologies and leveraging existing data more effectively to combat one of the world’s health challenges. The proposed framework not only enhances the current state of malaria detection but also opens new avenues for the application of similar techniques to other infectious diseases. Future work could be summarized as using alternate datasets to validate the proposed methodology and using other algorithms with fine-tuning mechanisms instead of transfer learning. Besides, this methodology can be generalized to other diseases such as leukemia, thereby contributing to a more scalable and effective approach to disease diagnosis through AI.

## Data Availability

The datasets analyzed during the current study are available in “Malaria Cell Images Dataset”, 2018. https://www.kaggle.com/datasets/iarunava/cell-images-for-detecting-malaria/data.
